# A novel descriptor based on atom-pair properties

**DOI:** 10.1186/s13321-016-0187-6

**Published:** 2017-01-05

**Authors:** Masataka Kuroda

**Affiliations:** Discovery Technology Laboratories, Innovative Research Division, Mitsubishi Tanabe Pharma Corporation, 1000 Kamoshida, Aoba-ku, Yokohama, 227-0033 Japan

**Keywords:** Atom-pair feature set, Property descriptor, Fingerprint, Pseudo-distance, Support vector machine

## Abstract

**Background:**

Molecular descriptors have been widely used to predict biological activities and physicochemical properties or to analyze chemical libraries on the basis of similarity. Although fingerprints and properties are generally used as descriptors, neither is perfect for these purposes. A fingerprint can distinguish between molecules, whereas a property may not do the same in certain cases, and vice versa. When the number of the training set is especially small, the construction of good predictive models is difficult. Herein, a novel descriptor integrating mutually compensating fingerprint and property characteristics is described. The format of this descriptor is not conventional. It has two dimensions with variable length in one dimension to represent one molecule. This format is not acceptable for any machine learning methods. Therefore the distance between molecules has been newly defined for application to machine learning techniques. The evaluation of this descriptor, as applied to classification tasks, was performed using a support vector machine after the features of the descriptor had been optimized by a genetic algorithm.

**Results:**

Because the optimizing feature is time-intensive due to the complicated calculation of distances between molecules, the optimization was forced to stop before it was completed. As a result, no remarkable improvement was observed in the classification results for the new descriptor compared with those for other descriptors in any evaluation set used in this work. However, extremely low accuracies were also not found for any set.

**Conclusions:**

The novel descriptor proposed in this work can potentially be used to make highly accurate predictive models. This new concept in descriptors is expected to be useful for developing novel predictive methods with quick training and high accuracy.

## Background

Several molecular descriptors have been developed to describe molecules as numbers [1–3]. They are usually used to predict biological activities towards proteins, which are called quantitative structure–activity relationships (QSAR) [4, 5], and physicochemical properties such as solubility or membrane permeability, which are called quantitative structure–property relationships (QSPR) [6]. They are also used to calculate the similarity of molecules for clustering or analysis of chemical libraries [7, 8].

Descriptors are broadly divided into two types. In the first type, numerical values represent the molecule as a whole, including physical properties such as molecular weight and octanol–water partition coefficient (logP). Although these properties can be measured, calculated values are generally used to predict the activities or properties of molecules. Other examples include the connectivity and shape indices developed by Kier and Hall [9, 10], which calculated from the two-dimensional structure. Structural energy and several other values can be calculated from the three-dimensional molecular configuration using the molecular orbital method [11]. Such descriptors are represented as numbers and facilitate the determination of relationships among molecules; they have widely been applied to QSAR and QSPR studies [6, 12].

In the second type of descriptor, the molecule is described as a list of its various components. The atom-pair descriptor, for example, includes the descriptions and connection information for two atoms as a single code [13]. Fingerprints are also widely used and treated as explicit codes according to their components. Extended-connectivity fingerprints (ECFP) are one such example, where the atoms connected within several bonds are encoded [14]. By contrast, graph kernels use a molecular graph in which atoms and bonds are replaced with nodes and edges, respectively [15–20]. They are treated implicitly and translated into other formats such as matrices so that machine learning techniques such as support vector machines (SVM) [21] can be used to predict activities or properties.

**Fig. 1:**
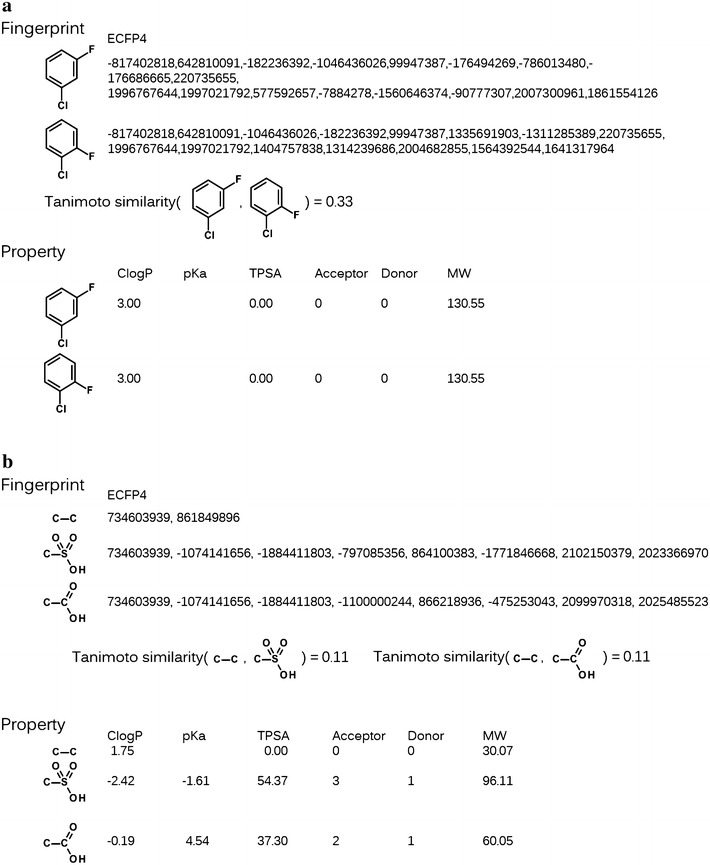
Examples illustrating the characteristics of property and fingerprint descriptors. For fingerprint, ECFP4 was calculated using the Pipeline Pilot software [22]; the numbers are shown as their own specific substructures. For properties, ClogP and pKa were calculated using the Daylight software [23] and the ChemAxon pKa plugin [24], respectively. **a** The fingerprint can distinguish between the relative positions of fluorine and chlorine atoms, but the property cannot. **b** The fingerprint shows that the difference between ethane and acetic acid is equal to that between ethane and methanesulfonate. By contrast, the property shows the difference between the acidic strengths of the two molecules

Figure 1 shows several simple examples that explain the characteristics of the fingerprint and property descriptors. The fingerprint used herein is ECFP4, as calculated using the BIOVIA Pipeline Pilot program [22]. The properties are ClogP, calculated using Daylight ClogP [23]; pKa, calculated using the ChemAxon pKa plugin [24]; topological polar surface area (TPSA) [25]; the numbers of hydrogen bond acceptors and donors; and molecular weight. Although the properties do not distinguish between halogen atoms at the ortho and meta positions, the Tanimoto coefficient [26] of the fingerprint does (Fig. 1a). By contrast, the Tanimoto coefficients for two acidic molecules against ethane are the same for a fingerprint, but the properties indicate the differences between the two molecules properly (Fig. 1b).

Generally, when a simple descriptor is used, different molecules happen to be represented by one description and correctly classifying molecules into active or non-active groups is impossible. By contrast, when a complicated descriptor with many and various features is used, the possibility that a molecule is described by a unique representation increases. However, the important features of the activity or property of the molecule are difficult to extract because highly similar parts are represented by different descriptions and the relationships between them may not be generated or may be buried in many irrelevant relationships. In this case, a large training set can generate good predictive models. In particular, at the beginning of drug discovery programs, activities are not available for a sufficient number of molecules to enable the generation of a good predictive model. If the features of fingerprints contain the relationships among them, they could compliment the lack of data and result in better predictive models.

To compensate for the lack of fingerprint and property characteristics, a new 2D descriptor has been developed. The basic concept is that the descriptor has (1) a small number of types of features, (2) numerically comparable features, (3) atom types described as features and (4) atomic locations. Although the new descriptor is similar to the atom-pair descriptor [13], it is represented as a list of feature sets for all heavy atom pairs. One feature set consists of four components, including the atom-type features for an atom, those for another atom, relationship features and isomerism features. This descriptor can be described as being written in two dimensions. For example, when a molecule is composed of twenty heavy atoms that make 380 atom pairs and the atom-pair descriptor is described as ten features, one pair can be a point in ten-dimensional space and this molecule can be characterized as 380 points.

Various machine learning methods have been often used for the prediction and virtual screening [27, 28]. For most of them, the input data have unique keys and their corresponding values; that is, they are linearly formatted. In this paper, SVM was chosen to evaluate the capability of the developed descriptor. For SVM to be used, the interface for loading the input data and the method for generating the predictive model should be modified or developed for the novel two-dimensionally formatted descriptor. Hence, the distance between molecules was newly defined using the descriptor and was converted into the kernel matrix loaded by general SVM programs. The features should be correctly adjusted to predict the activities or properties because there are no relationships among these features. No attempt was made to find common weights for features because of the unavailability of a good global training set to determine them. However, suitable weights for each training set were searched using the genetic algorithm (GA) [29] in the evaluation.

## Materials and experimental methods

### Generation of descriptor

**Fig. 2:**
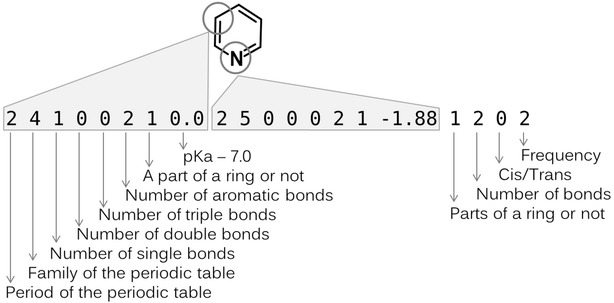
Illustration of the one atom-pair feature set. The *eight features* represent the atom types of the carbon and nitrogen atoms in the *circles*, respectively. The *next two features* show the relationship between the two atoms. The *last feature*, which is *nineteenth column*, shows isomerism. The *final column* is the frequency of the grouped atom-pair feature sets

The novel descriptor is described as a series of lists of atom-pair feature sets. Each atom-pair feature set consists of four components: the atom type for an atom ($atom_{i}$), that for another atom ($atom_{j}$), the relationship between the two atoms, and their isomerism, as shown in the example in Fig. 2.

**Fig. 3:**
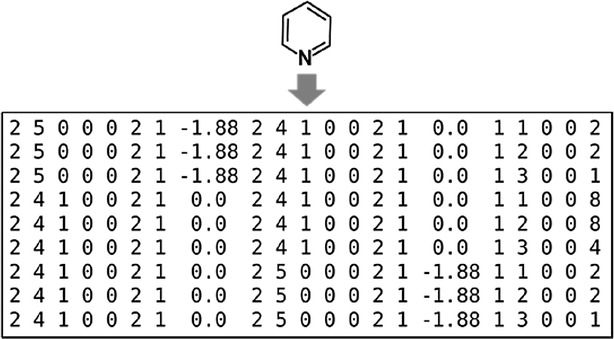
An example of the full description of pyridine generated by the new atom-pair feature definition

The atom type contains eight features: the periodic period and family, the number of single bonds, the number of double bonds, the number of triple bonds, the number of aromatic bonds, the flag for a part of a ring and pKa. Drug-like compounds are mainly treated here, and the transition elements are ignored. The pKa values were calculated using the ChemAxon pKa plugin [24] and 7.0 was subtracted from the calculated value. For atoms whose pKa values are not included in the software, the pKa feature was set to 0. The relationship includes two features: a flag for parts of a ring and the number of bonds in the shortest path between the two atoms. Isomerism is the cis–trans configuration, which is calculated when the following conditions are matched: (1) The number of bonds between the two atoms is three or more. (2) The orders of the bonds between $atom_{i}$ and $atom_{i + 1}$, between $atom_{i + 1}$ and $atom_{i + 2}$ and between $atom_{i + 2}$ and $atom_{i + 3}$ are single, double and single, respectively, and neither of the four atoms is aromatic. If $atom_{i}$ and $atom_{i + 3}$ take the cis position, as judged from their 2D coordinates, this feature is set to −1. If the atoms are in the trans form, it is set to 1. Otherwise, it is set to 0. The total number of features is nineteen per atom pair. The atom-pair features for all heavy atom pairs are calculated. The same atom-pair feature sets are grouped together, and the frequency of the set is added as the twentieth column. The frequency is not treated as one of features in this study. The sets are individually represented as rows, as illustrated in Fig. 3.

The program was written in Perl without any chemical toolkits.

### Evaluation

Eleven experimental datasets used to evaluate the new descriptor are summarized in Table 1.

**Table 1:** Numbers of positive and negative samples used for the evaluation

Dataset name	Positive samples	Negative samples
MUTAG	125	63
PTC-MM	129	207
PTC-FM	143	206
PTC-MR	152	192
PTC-FR	121	230
BBB	276	139
BIO	159	106
BZR	157	149
COX2	148	155
DHFR	124	269
ER	181	265

The MUTAG dataset contains 188 aromatic and heteroaromatic nitro compounds tested for mutagenicity [30]. The predictive toxicology challenge (PTC) dataset is composed of four carcinogenicity sets clinically tested for male mice (PTC-MM), female mice (PTC-FM), male rats (PTC-MR) and female rats (PTC-FR) [31]. It includes 226 compounds in PTC-MM, 349 compounds in PTC-MF, 344 compounds in PTC-MR and 351 compounds in PTC-FR. The BBB dataset, consisting of 415 compounds, is used for the blood–brain barrier (BBB) penetration test [32]. The BIO dataset addresses the human oral bioavailability of 265 compounds [33]. The BZR, COX2, DHFR and ER sets contain 306 compounds that exhibit benzodiazepine receptor (BZR) activity, 303 compounds that exhibit cyclooxygenase-2 (COX-2) activity, 393 compounds that exhibit dihydrofolate reductase (DHFR) activity and 446 compounds that exhibit estrogen receptor (ER) activity, respectively, as provided by Sutherland et al. [34]. These data sets were originally used to evaluate the atom environment kernel reported by Yamashita et al. [15] and were exchanged through personal communication. Each data set was randomly split into the training set, which included 90% of the components, and the test set, which included 10% of the components. Twenty sets were generated for one experimental datum by repeating the splitting procedure. Predictive-model building and prediction were performed for each set.

SVM was used as the predictor, and the kernel matrix was prepared from the pseudo-distances between molecules. The weights of the features were optimized using GA. The area under the receiver operating characteristic curve (AUC) was calculated to enable a comparison of the accuracy of prediction.

### Pseudo-distance calculation

The pseudo-distance was calculated as the root mean square of the two distances from a molecule, A, to another one, B, and from B to A. Let ${\mathbf{A}} = \left( {\varvec{a}_{1} , \ldots ,\varvec{a}_{na} } \right)$ and ${\mathbf{B}} = \left( {\varvec{b}_{1} , \ldots ,\varvec{b}_{nb} } \right)$ be the atom-pair feature sets of A and B except their frequencies, respectively, where *na* and *nb* are the numbers of the feature sets. The equation is written as follows:

1 $$d_{AB} = \sqrt {d_{{\overrightarrow {{{\mathbf{AB}}}} }}^{2} + d_{{\overrightarrow {{{\mathbf{BA}}}} }}^{2} } ,$$

where $d_{{\overrightarrow {{{\mathbf{AB}}}} }}$ is the distance from **A** to **B**, defined as the root mean square of the distances from all feature sets of **A** to **B**. The equations are written as

2 $$d_{{\overrightarrow {{{\mathbf{AB}}}} }} = \sqrt {\frac{{\mathop \sum \nolimits_{i}^{na} f\left( {\varvec{a}_{i} , \varvec{B}} \right)^{2} }}{{n_{A} }}}$$

and

3 $$f\left( {\varvec{a}_{i} , \varvec{B}} \right) = n_{{a_{i} }} \cdot \mathop {\hbox{min} }\limits_{{\varvec{b} \in \varvec{B}}} \varvec{d}_{{\varvec{a}_{i} \varvec{b}}} ,$$

where $n_{A}$ is the total number of atom pairs of molecule A, $n_{{a_{i} }}$ is the frequency of the set, $\varvec{a}_{i}$, and $\varvec{d}_{{\varvec{a}_{i} \varvec{b}}} = \left( {d_{{\varvec{a}_{i} \varvec{b}_{1} }} , \ldots ,d_{{\varvec{a}_{i} \varvec{b}_{nb} }} } \right)$; $d_{{\varvec{a}_{i} \varvec{b}_{j} }}$ is the Euclidean distance calculated using the features of the set, written as

4 $$d_{{\varvec{a}_{i} \varvec{b}_{j} }} = \left| {\varvec{a}_{i} - \varvec{b}_{j} } \right| .$$

If the frequency of an atom-pair feature set is not considered, the distance in this definition may be misunderstood because it does not depend on the frequencies of the feature sets, $\varvec{a}_{i}$ and $\varvec{b}_{j}$. To avoid this problem, Eq. (3) is modified as follows:

1. The summed distance parameter, *d*, is initialized to be 0 under the assumption that $n_{{a_{i} }}^{{\prime }} = n_{{a_{i} }}$.
2. An atom-pair feature set of **B**, $\varvec{b}_{m}$, is identified such that the distance from $\varvec{a}_{i}$ is the smallest for **B**.
3. If $n'_{{a_{i} }}$ is the same as or less than $n_{{b_{m} }}$, which is the frequency of $\varvec{b}_{m}$, the distance is calculated as follows, and the process is finished:
  5 $$d = d + n_{{a_{i} }}^{{\prime }} d_{{\varvec{a}_{i} \varvec{b}_{m} }}$$

Otherwise, if $n_{{a_{i} }}^{{\prime }}$ is larger than $n_{{b_{m} }}$, $n_{{b_{m} }}$ is used in Eq. (5) instead of $n'_{{a_{i} }}$, leading to the equations

6 $$d = d + n_{{b_{m} }} d_{{\varvec{a}_{i} \varvec{b}_{m} }}$$

7 $$n'_{{a_{i} }} = n'_{{a_{i} }} - n_{{b_{m} }}$$

and $\varvec{b}_{m}$ is removed from **B**.

4. Steps 2 and 3 are repeated.

If $n_{{a_{i} }}$ is larger than $n_{B}$, that is, if all atom-pair feature sets of **B** are gone before $n'_{{a_{i} }}$ becomes zero in the iteration, *d* is adjusted according to the ratio of both numbers as:

8 $$d = d \cdot \frac{{n_{{a_{i} }} }}{{n_{B} }}$$

### Classification by SVM

To translate from the pseudo-distance matrix into the kernel matrix, a Gaussian function was used:

9 $$f\left( d \right) = { \exp }\left( {\frac{{ - d^{2} }}{\gamma }} \right)$$

The parameter $\gamma$ should be determined properly. The matrixes were calculated for the parameter gamma from $\left\{ {e^{i} |i \in \left\{ { - 5, - 4, - 3, - 2, - 1, 0, 1, 2, 3, 4, 5} \right\}} \right\}$ which was enough to include the proper value at first. The numerical range for this parameter in the preliminary experiments was roughly searched in the feature optimization described in the next section using a PTC-FM set for the evaluation. The default range was finally defined as $e^{ - 3} \le \gamma \le e^{3}$. However, this range should be adjusted because the distance distribution of a molecular set depends on the molecules belonging to the set. Let ***M*** be all molecules and $\gamma$ be selected from $$\left\{ {\begin{array}{*{20}l} {e^{s \cdot i + t} |t = \hbox{min} \left\{ { - 3, \left\{ {\ln \left( {d_{AB} } \right)|A \in \varvec{M},B \in \varvec{M},A \ne B} \right\}} \right\},} \hfill \\ {\quad \quad \quad s = \frac{{\hbox{max} \left\{ {3, \left\{ {\ln \left( {d_{AB} } \right)|A \in \varvec{M},B \in \varvec{M},A \ne B} \right\}} \right\} - t}}{9},} \hfill \\ {\quad \quad \quad \quad \quad i \in \left\{ {0, 1, 2, 3, 4, 5, 6, 7, 8, 9} \right\}} \hfill \\ \end{array} } \right\}.$$

LIBSVM was used for the SVM solver [28, 35, 36]. The parameter of the constraints-violation cost, C, in LIBSVM was chosen from $\left\{ {2^{n} |n \in \left\{ { - 5, - 4, - 3, - 2, - 1, 0, 1, 2, 3, 4, 5} \right\}} \right\}$. The best parameter set was found by an exhaustive search against the two parameters.

### Feature optimization

GA was applied to optimize the weights of the features [29]. The two atoms in the atom-pair feature set have the same types of features, and the same set of weights was applied to the features of the two atoms. Therefore, the number of weights requiring optimization was reduced from nineteen to eleven. The population size was set to 32. The probabilities of mutation and crossover were 0.15 and 0.8, respectively. The number of generations was set to 20 because of the long calculation time. Generally, the number of generations used here is too small to obtain fully optimized weights. To calculate complete evaluation sets in a realistic time, weights were roughly searched in the preliminary runs using a PTC_FM set before the start of the evaluation and the resulting weights were set as the initial ones for all evaluation sets. The weights were increased in the range from −0.1 to 0.1 from those at the previous iteration of GA in each evaluation set.

For each genome set, 1000 new training and validation sets were generated from the original training set using the bagging method. Each set was classified by SVM using the aforementioned newly defined distance. The evaluation function in GA was set to have the averaged prediction accuracy from the 1000 sets.

Optimization by GA always yielded several weight sets with identical scores. To select the best weight set, cross validations on LIBSVM were performed against all sets. The predictive model with the best result was applied to the test set.

GA and LIBSVM were implemented in C++. The optimization tasks were computed using 16 cores in parallel on two Intel Xeon E5-2690 2.9 GHz cpus.

### Classification using molecular properties

The properties used in this study were ClogP [23], acidic pKa1, acidic pKa2, basic pKa1, acidic pKa2 [24], TPSA [25], number of hydrogen-bond acceptors, number of hydrogen-bond donors, molecular weight, molecular refractivity (MR) [37], number of rotatable bonds and 2D descriptors calculated using the Molecular Operating Environment (MOE) software [38]. Properties whose standard deviations were less than 0.01 or whose correlation coefficients against another property were less than −0.9 or greater than 0.9 were not used for the training. The properties used for training were normalized. The predictions were performed using LIBSVM. Two parameters, the penalty of the error term and γ, were unknown before training; the grid search was conducted in the tenfold cross-validation mode to identify the best parameter set.

## Results and discussion

**Fig. 4:**
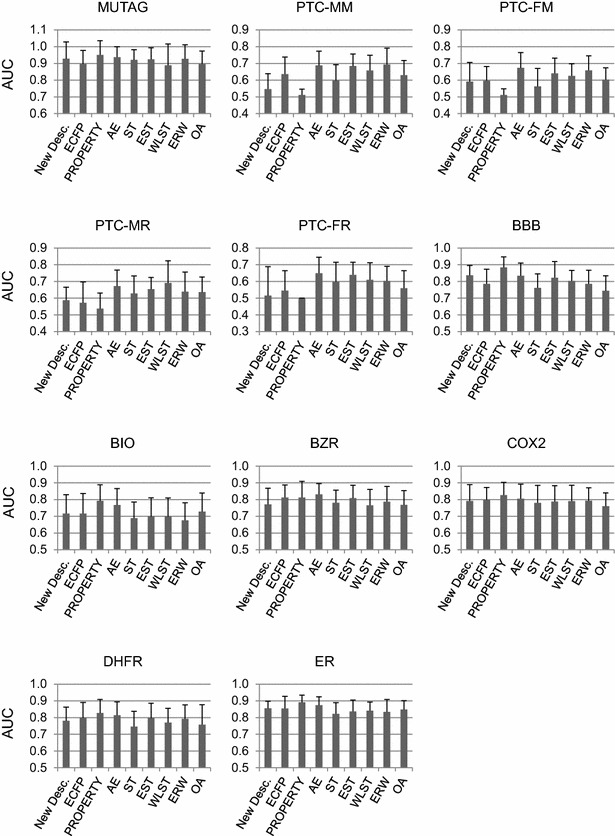
Prediction accuracies of the new descriptor and other descriptors (ECFP, PROPERTY, AE, ST, EST, WLST, ERW, OA). The classifications for ECFP, AE, ST, EST, WLST, ERW and OA were performed by Yamashita et al. [15]

The classification results by SVM for the developed descriptor, ECFP [14], the property descriptor and graph kernels (AE [15], ST [16], EST [17], WLST [18], ERW [19] and OA [20]) are summarized in Fig. 4. For all sets except PTCs and BZR, the classifications using molecular properties are the best descriptors. For the four PTC sets, not all scores for the classification of test sets were calculated normally. These results show that the property descriptor cannot obviously or completely represent the structure for any data set. For the new descriptor, BBB exhibits the best AUC, except in the case of the property descriptor. For the other sets, the AUCs are not the worst. However, the results of the four PTC sets are relatively poor. The varieties of compounds in the four PTC sets are relatively larger than those in the other sets.

The averaged weights for the evaluation sets after the optimizations are listed in Table 2. The weights for all sets are not significantly different from the initial weights because the small number of generations (20) was not sufficiently optimized by GA. Furthermore, the automatic translation of pseudo-distances to matrices by a Gaussian function might yield very small numbers for the long distances, which, in turn, may be ineffective in extracting the activity information between molecules. As a result, the prediction accuracies become worse than those of other evaluation sets. The calculation time from the weight optimization to the prediction for one evaluation set varied from 1 day and 13 h for MUTAG to 3 days and 8 h for DHFR. If more cpu cores or gpus are used, the number of iterations in the optimization can be increased in a realistic time and the prediction accuracy could be improved. Given the number of generations in the feature optimization using GA in this study, it is noted none of the evaluation sets have the worst results and one of them, in particular, shows almost the best results. The newly developed descriptor has potential for accurate prediction.

**Table 2:** Averaged weights after the GA optimization for each evaluation set

Feature	Initial weight^a^	MUTAG	PTC-MM	PTC-FM	PTC-MR	PTC-FR	BBB	BIO	BZR	COX2	DHFR	ER
Period	0.927	0.869	0.912	0.907	0.885	0.925	0.947	0.893	0.866	0.860	0.899	0.878
Family	0.400	0.408	0.436	0.359	0.337	0.353	0.460	0.418	0.460	0.426	0.355	0.393
Single bonds	0.370	0.323	0.329	0.354	0.286	0.325	0.369	0.439	0.361	0.312	0.301	0.359
Double bonds	0.013	0.055	0.061	0.068	0.045	0.052	0.058	0.056	0.055	0.071	0.049	0.056
Triple bonds	0.504	0.519	0.527	0.490	0.491	0.535	0.510	0.498	0.477	0.491	0.495	0.479
Aromatic bonds	0.931	0.925	0.923	0.931	0.965	0.906	0.886	0.874	0.878	0.924	0.927	0.910
Part of a ring	0.340	0.303	0.317	0.361	0.387	0.360	0.357	0.336	0.417	0.316	0.341	0.338
pKa	0.688	0.650	0.688	0.689	0.707	0.705	0.660	0.687	0.696	0.703	0.645	0.719
Parts of a ring	0.264	0.307	0.242	0.267	0.292	0.250	0.264	0.284	0.299	0.274	0.311	0.281
Bonds between the atoms	0.013	0.098	0.053	0.045	0.073	0.054	0.088	0.051	0.102	0.108	0.106	0.096
cis/trans	0.925	0.945	0.900	0.909	0.890	0.907	0.936	0.921	0.927	0.919	0.910	0.913

^a^The weights were roughly calculated using a PTC_FM set before the evaluation was started

**Fig. 5:**
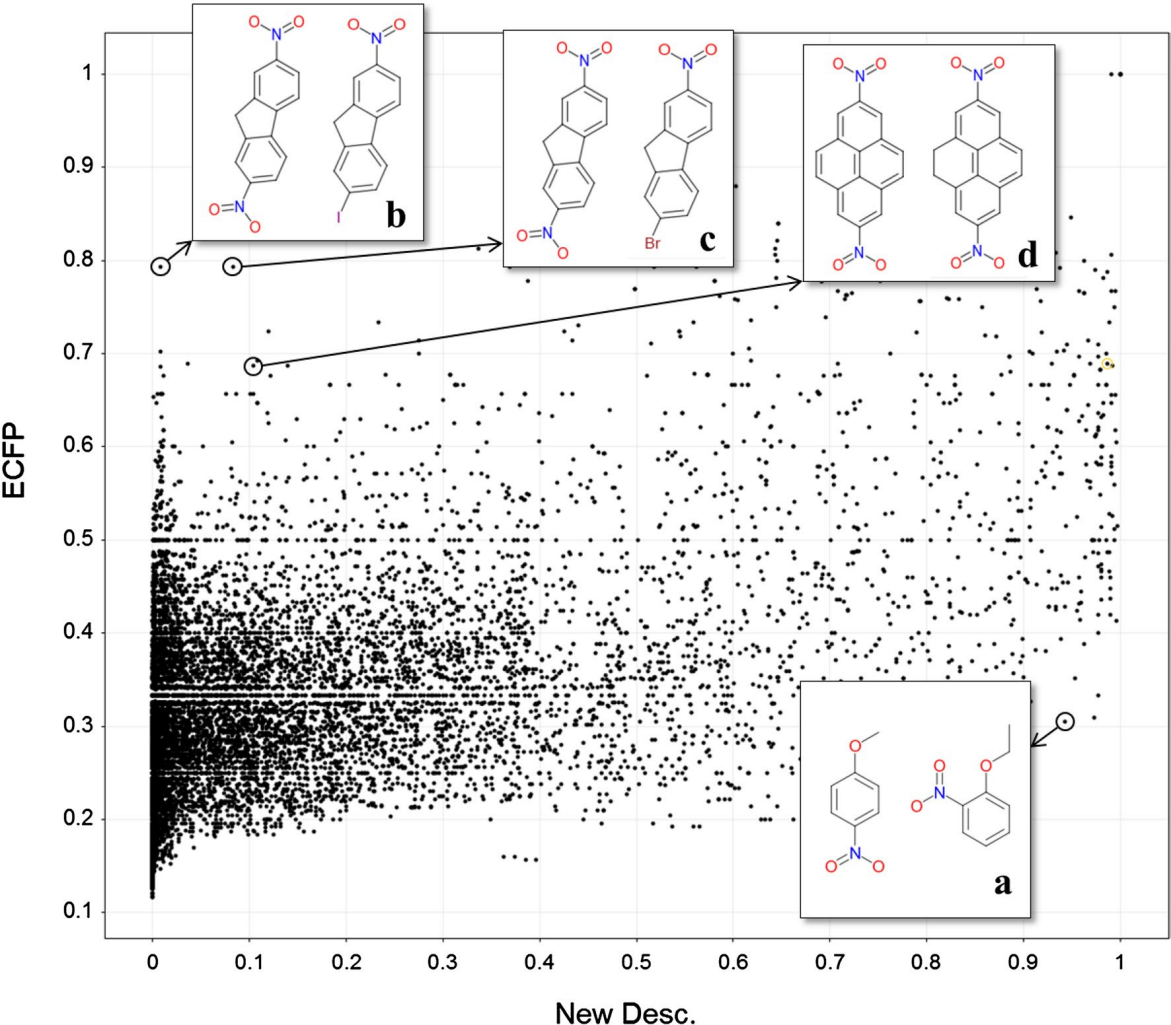
The relationship of molecular similarity between the new descriptor and ECFP in a training set of the MUTAG set. The values of the kernel matrix after the optimization and the Tanimoto coefficient between two molecules are used for the new descriptor and ECFP, respectively

**Fig. 6:**
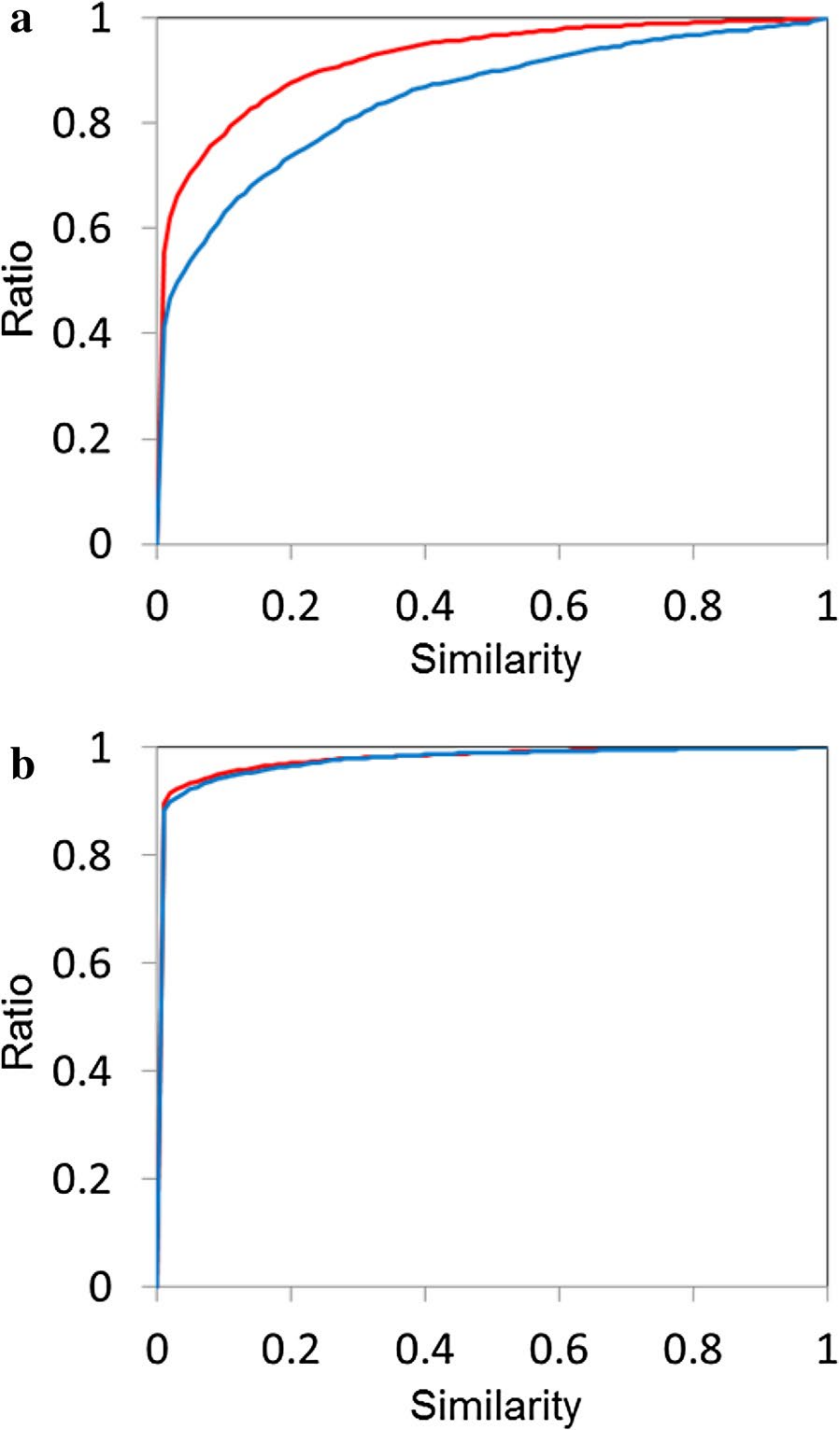
Comparison of the ratio accumulation curves for similarity of any molecular pairs for the same labels (positive–positive or negative–negative) in *blue* and the different labels (positive–negative or negative–positive) in *red*. The values of the kernel matrix after the optimization and the Tanimoto coefficient are used for the new descriptor and ECFP, respectively. **a** The training set of the MUTAG set used in Fig. 5. **b** A training set of the PTC-FM set

The feature selection is generally carried out to take relevant features from the huge number of features [3, 39]. In this study, the weight determination by GA can be the feature selection. The weight of the irrelevant feature becomes lower relatively. Table 2 reveals some interesting information regarding the features. The weights of the period, the number of aromatic bonds, pKa and the cis/trans flag show larger numbers, indicating that these features may be equally important for the activity classification. By contrast, the weights of the number of double bonds and the number of bonds between two atoms are less than 0.1. The number of bonds between two atoms appears to be irrelevant, but it varies from one to greater than ten. It might reach a balance between feature sets at long and short distances. The weight of the period is approximately twice as large as that of the family. Thus, the difference between the atomic neighbours of a period is nearly the same as that of atoms that belong to the same family and are separated by two periods. For example, the difference between carbon and nitrogen atoms may be the same as that between fluorine and bromine atoms. Full feature optimization could lead to a clearer understanding of which features are quantitatively important. Figure 5 shows the relationship between the new descriptor and ECFP for a training set of the MUTAG set which results the best performance for the new descriptor set. Each dot indicates the value of the kernel matrix after the optimization and the Tanimoto coefficient between two molecules for the new descriptor and ECFP, respectively. The novel descriptor tends to show the higher similarity than ECFP for relatively small molecules (Fig. 5a). On the other hand, bromine and iodine atoms lead to dissimilarity of molecules (Fig. 5b, c) and the alicyclic ring seems to be more sensitive than the aromatic ring against the similarity (Fig. 5d). Figure 6 shows the ratio accumulation curves of the same labels and different labels that the two compounds of a pair have. In Fig. 6a, the line in red for the different labels runs above that in blue for the same labels, that is, the pseudo-distance with the new descriptor distinguished the molecular pairs of the same labels from those of the different labels for the MUTAG set. In Fig. 6b, on the other hand, there is little difference between the two curves for the PTC-FR set which shows the worst accuracy. In this case, the pseudo-distance failed to catch the important information linking to the activity difference.

The conversion from the descriptor to the distance might bury the relevant activity information, as previously mentioned. I first tried to generate a decision tree from the training set because the decision tree was thought to be free from the feature optimization performed for SMV. Additionally, it matched the concept of the new descriptor, which contains numerical values to enable a comparison of the same feature in various molecules. To construct a decision tree, samples in the training set are sorted according to the values of a selected feature and the branch point is determined to achieve large separation between classes [40]. In general, this operation is performed repeatedly and the classification is successfully completed. However, the variety of values contained in each feature, except pKa, is very small. In the MUTAG set, for example, the number of varieties of values contained in the period and family features are only four, whereas that contained in the connected-bonds feature is fourteen, which is the maximum number among all features except pKa. Furthermore, because there are more than one atom-pair feature sets in a molecule, that is, all features of a molecule have more than one value, the molecules cannot be sorted primarily against one feature, as done in the conventional decision-tree-making process. Although a couple of branching methods were tested, the branching procedure stopped before the classification was completed or huge numbers of branches were generated.

Recently, two deep neural network methods based on atom-pair features have been reported [41, 42]. One translates into a fingerprint [41], and the other uses graph convolutions [42]. The convolution procedure is basically performed against the connected atoms in both methods. The deep neural network could be applied to the new descriptor, which is itself described as nineteen-dimensional data instead of convolution along the atom connections, and automatically makes the descriptor selection during the training. This approach is expected to be an alternative prediction method.

## Conclusions

This article presents a novel descriptor based on the atom-pair property. Features of types other than those used here can be added easily. Although chiral information was not used in this study, the chirality often affects the activity significantly and should be contained in the descriptor. This novel descriptor shows the possibility of constructing a predictive model with greater accuracy, although the optimization and parameter determinations for the Gaussian function were not sufficient in this study. The generation of the predictive model described in this paper requires substantial time (1.5 days at least for 20 generations in GA). It would be difficult to use this predictive procedure in the drug-research programs at this moment. Hence, novel prediction methods with faster training will be developed in future work. In addition to the deep neural network mentioned above, for example, the decision tree is still an attractive method for this descriptor. If the branching procedure is contrived to complete the classification, the training time will be extremely shorter than that by the GA + SVM method used in this study.
